# NT-proBNP as a Biomarker in MGUS and Multiple Myeloma: A Retrospective Analysis

**DOI:** 10.3390/jcm14186381

**Published:** 2025-09-10

**Authors:** Jooheon Park, Yong Jun Choi, Ha Jin Lim, Yong Jun Kwon, Myung Geun Shin, Eun-Hee Nah

**Affiliations:** 1 Department of Laboratory Medicine, Chonnam National University Hwasun Hospital, Hwasun 58128, Republic of Koreaazarsis@hanmail.net (Y.J.C.); 2 Department of Laboratory Medicine, Chonnam National University Hospital, Gwangju 61469, Republic of Korea; hajin00905@naver.com (H.J.L.); garei09@gmail.com (Y.J.K.)

**Keywords:** NT-proBNP, monoclonal gammopathy of undetermined significance, multiple myeloma, disease severity, β2-microglobulin, staging

## Abstract

**Background**: N-terminal pro-B-type natriuretic peptide (NT-proBNP) is a well-established biomarker of cardiac stress and has recently been implicated in hematologic malignancies. However, research on how NT-proBNP changes from monoclonal gammopathy of undetermined significance (MGUS) to multiple myeloma (MM), and its association with disease severity and progression, remains limited. This study evaluated whether NT-proBNP levels are associated with disease severity and progression in patients with MGUS and MM. **Methods**: This retrospective cross-sectional study included 121 patients with MGUS and 472 patients with MM. MGUS risk was stratified based on the presence of three major risk factors, while MM was staged according to the ISS, R-ISS, and R2-ISS systems. Associations between NT-proBNP and clinical or laboratory parameters were evaluated using univariate and multivariate regression. **Results**: NT-proBNP levels did not significantly differ between the MGUS and MM groups. In MGUS, NT-proBNP levels were positively associated with β2-microglobulin (*p* = 0.018) and creatinine (*p* < 0.001). In MM, NT-proBNP levels increased with advancing disease stage in all staging systems (*p* < 0.001), but these associations were no longer significant in multivariate models. Instead, β2-microglobulin, LDH, creatinine, and albumin remained independently associated with NT-proBNP levels. **Conclusions**: NT-proBNP levels were not associated with MGUS risk factors and showed limited value for risk stratification in MGUS. In MM, NT-proBNP may reflect disease burden but lacks independent value as a marker of disease stage. NT-proBNP may serve as an indicator of overall disease burden, but has limited value as an independent biomarker for disease severity in MM.

## 1. Introduction

N-terminal pro-B-type natriuretic peptide (NT-proBNP) is primarily secreted by ventricular myocardium in response to increased wall stress and is widely used as a biomarker for cardiac dysfunction [1]. Elevated NT-proBNP levels have demonstrated prognostic utility for cardiovascular events and mortality in patients with heart failure [2]. More recently, NT-proBNP elevations have also been reported in various malignancies, including hematologic cancers [3,4]. Notably, markedly elevated NT-proBNP levels have been observed in hematologic malignancies even in the absence of overt cardiac dysfunction [5]. Natriuretic peptides may not only reflect myocardial injury but also be produced directly by malignant cells. In oncology, elevated NT-proBNP levels have been associated with greater tumor burden, disease progression, and poorer overall survival [6]. In the context of multiple myeloma (MM), Milani et al. suggested that NT-proBNP serves as a simple and effective prognostic biomarker for survival [7], and other studies have linked its elevation to disease severity [8].

MM is typically preceded by monoclonal gammopathy of undetermined significance (MGUS), an asymptomatic premalignant condition characterized by low serum monoclonal (M) protein levels and a small proportion of clonal plasma cells in the bone marrow [9]. MGUS progresses to MM or other plasma cell disorders, including Waldenström macroglobulinemia (WM) and amyloid light-chain (AL) amyloidosis, at an annual rate of approximately 1% [10]. Despite its clinical relevance, large-scale studies evaluating NT-proBNP levels specifically in MGUS patients remain limited. Some research has focused on AL amyloidosis, which may arise from MGUS and often involves cardiac deposition, with NT-proBNP serving as a key prognostic biomarker [11]. In MGUS, elevated NT-proBNP may signal underlying cardiac amyloidosis even in the absence of apparent cardiac disease and has been shown to predict survival independently of age and comorbidities [12].

However, data regarding the natural trajectory of NT-proBNP and its association with disease severity or progression from MGUS to MM are scarce. Therefore, the aim of this study is to investigate whether NT-proBNP levels are associated with disease severity and progression in patients with MGUS and MM.

## 2. Materials and Methods

### 2.1. Study Design and Data Collection

This was a cross-sectional, retrospective study including patients diagnosed with MGUS or MM between January 2014 and December 2023 at two university hospitals. Patients were diagnosed according to the IMWG definition of MM [10]. Inclusion criteria were: (1) confirmed diagnosis of MGUS or symptomatic MM, (2) age > 18 years, and (3) at least one serum NT-proBNP measurement obtained before initiation of chemotherapy. Patients who declined the use of their medical records were excluded. Data were collected using the Chonnam National University Hospital Information System. Laboratory results were extracted from the laboratory information system, and clinical data were obtained from the Clinical Data Warehouse and electronic medical records. Collected laboratory data included: M-protein type and concentration, serum immunofixation electrophoresis (IFE), complete blood count (CBC), albumin, lactate dehydrogenase (LDH), creatinine, uric acid, calcium, β2-microglobulin (B2M), NT-proBNP, immunoglobulins (IgG, IgA, IgM), serum free light chain (FLC) levels and κ/λ ratio, and cytogenetic data.

The study was approved by the Institutional Review Board of Chonnam National University Hospital (CNUH-2025-237). Patient consent was waived due to the retrospective nature of the study.

### 2.2. MGUS Risk Stratification and MM Staging System

MGUS risk stratification was based on the Mayo Clinic model, which includes three major risk factors: abnormal serum FLC ratio, non-IgG isotype, and serum M-protein concentration ≥ 1.5 g/dL. Patients with all three risk factors were classified as high-risk MGUS. Those with two, one, or none of the risk factors were classified as high-intermediate, low-intermediate, and low-risk MGUS, respectively [13,14].

MM staging was determined using the International Staging System (ISS), the Revised ISS (R-ISS), and the Second Revised ISS (R2-ISS). The ISS is based on serum B2M and albumin levels. The R-ISS incorporates ISS stage, high-risk cytogenetics, and LDH level. The R2-ISS integrates ISS, LDH, and additional cytogenetic abnormalities [15,16,17].

### 2.3. Laboratory Measurements

Venous blood samples were collected after overnight fasting. Serum was separated by centrifugation at 3000 rpm for 10 min. NT-proBNP levels were measured using the Cobas e 601 analyzer (Roche Diagnostics, Indianapolis, IN, USA), with a measuring range of 5–35,000 pg/mL. Values exceeding the upper range were measured up to 70,000 pg/mL (8260 pmol/L) after 2-fold dilution. The assay showed a coefficient of variation (CV) of 2.7–3.1%. Serum B2M levels were measured using the Architect System (Abbott Laboratories, IL, USA), with a detection range of 0.250–16.000 mg/L and CV < 6%.

### 2.4. Statistical Analysis

All analyses were conducted using SAS version 9.4 (SAS Institute, Cary, NC, USA). Continuous variables were expressed as median and interquartile range (IQR), and categorical variables as frequency and percentage. Non-normally distributed numerical variables were compared using the Wilcoxon rank-sum test or Kruskal–Wallis test, with post hoc pairwise comparisons based on the Dwass-Steel-Critchlow-Fligner method. Categorical variables were compared using the chi-square or Fisher’s exact test. Associations between NT-proBNP and disease stage or laboratory parameters were evaluated using univariate and multivariate regression analyses, adjusting for age, sex, and relevant laboratory data.

## 3. Results

### 3.1. Characteristics of the Study Subjects

**Figure 1:**
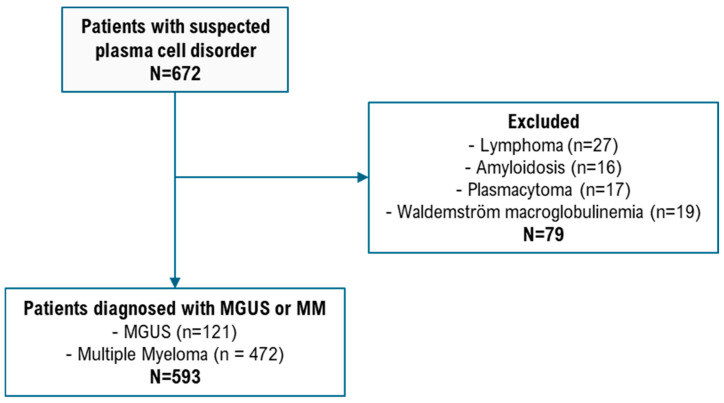
Flowchart of participant selection.

A total of 121 patients with MGUS and 472 patients with MM were included (Figure 1). The detailed characteristics of the study cohort are presented in Table 1. The median age of the subjects with MGUS and MM were 73.0 years (IQR = 69–78) and 70.0 years (IQR = 64–76), respectively. Among MGUS patients, 86 (71.1%) were male, while in the MM group, 249 (52.8%) were male. The median NT-proBNP level was 421 pg/mL (IQR 98–2753) in MGUS and 320.5 pg/mL (IQR 113.7–1104.5) in MM. There were statistically significant differences between patients with MGUS and those with MM in terms of age, sex, M-protein, non-IgG isotype percentage, B2M, abnormal FLC ratio, LDH, hemoglobin, platelet count, and cytogenetic abnormalities. Compared to MGUS patients, those with MM were younger and more likely to be female (*p* < 0.001). MM patients also had higher levels of M-protein, a greater proportion of non-IgG isotypes, elevated B2M, more frequent abnormal FLC ratios, and more cytogenetic abnormalities. LDH, hemoglobin levels, and platelet counts were lower in MM patients (*p* < 0.001). There was no significant difference in NT-proBNP levels between the MGUS and MM groups (Table 1).

### 3.2. MGUS Characteristics by Risk Group

There were statistically significant differences among MGUS risk groups in the levels of M-protein, the proportion of non-IgG isotype, and the frequency of abnormal FLC ratio (*p* < 0.001). However, there was no significant difference in NT-proBNP levels among the MGUS groups stratified according to the Mayo Clinic risk model (Table 2).

### 3.3. Factors Associated with NT-proBNP in MGUS

There were no correlations between NT-proBNP levels and the three major MGUS risk factors: abnormal serum FLC ratio, non-IgG isotype, and serum M-protein concentration ≥ 1.5 g/dL. In univariate regression analysis, NT-proBNP levels in MGUS patients were positively associated with B2M and creatinine, and negatively associated with albumin and hemoglobin levels. In multivariate analysis, B2M (*p* = 0.018) and creatinine (*p* < 0.001) remained significantly associated with NT-proBNP levels (Table 3).

### 3.4. NT-proBNP and MM Staging

NT-proBNP levels increased with advancing MM stage, a trend consistently observed across the ISS, R-ISS, and R2-ISS staging systems. Patients with more advanced MM stages were older (*p* < 0.001) and more likely to be male (*p* = 0.047). There were statistically significant differences among MM staging groups in NT-proBNP levels, M-protein concentration, proportion of non-IgG isotypes, and levels of B2M, creatinine, LDH, hemoglobin, and platelet count (*p* < 0.001). B2M, creatinine, and LDH levels increased with stage, whereas albumin, hemoglobin, and platelet count decreased. Cytogenetic analysis revealed a higher frequency of 1q amplification in patients with advanced-stage MM (Table 4).

### 3.5. Factors Associated with NT-proBNP Levels in MM

In univariate analysis, NT-proBNP levels in MM were positively associated with ISS stage III (as well as R-ISS stage III and R2-ISS stages III and IV), B2M, LDH, and creatinine, and negatively associated with serum albumin. However, in multivariate analysis, the association with MM stage was no longer significant. Instead, B2M (except in the ISS model), LDH, creatinine, and albumin remained independently associated with NT-proBNP levels (Table 5).

## 4. Discussion

This study demonstrates that NT-proBNP levels are not associated with established MGUS risk factors—including abnormal serum FLC ratio, M-protein ≥ 1.5 g/dL, or non-IgG isotype—indicating its limited utility in MGUS risk stratification. In contrast, NT-proBNP levels in MM were significantly correlated with markers of disease burden such as B2M, LDH, creatinine, and albumin, though they were not independently associated with disease stage after adjusting for these variables. These findings suggest that NT-proBNP reflects disease burden but lacks utility as a standalone biomarker of disease severity in MM.

MGUS is an asymptomatic, premalignant clonal plasma cell disorder and the recognized precursor of several lymphoplasmacytic malignancies, including MM. It is defined by low levels of serum M-protein and a small proportion of clonal plasma cells in the bone marrow. In this study, NT-proBNP levels did not significantly differ between patients with MGUS and MM, despite MM patients exhibiting higher levels of M-protein, more frequent non-IgG isotypes, elevated B2M, and a greater prevalence of abnormal FLC ratios. Cytogenetic alterations are considered key events in the transformation of MGUS to malignant disease. The acquisition of specific genetic abnormalities is believed to influence whether MGUS remains indolent or progresses to overt plasma cell malignancy [18,19]. Although genomic profiling has become integral in MM risk stratification [20,21,22], its application in MGUS has been limited. In the present study, the relationship between NT-proBNP and cytogenetic alterations could not be assessed due to insufficient data.

While direct evidence regarding NT-proBNP in MGUS is sparse, studies in AL amyloidosis—a disease that often originates from MGUS—have shown that NT-proBNP is a sensitive and specific marker of cardiac involvement and prognosis [11]. These findings raise the possibility that NT-proBNP might serve as an early marker for subclinical cardiac involvement even at the MGUS stage. However, in the current study, NT-proBNP levels did not significantly differ among MGUS risk groups defined by the Mayo Clinic model. Multivariate analysis revealed that NT-proBNP levels in MGUS patients were independently associated with B2M and creatinine. B2M reflects tumor burden, renal function, and immune activation [23,24,25], while its elevation in kidney injury underscores its role as a renal marker [26,27].

Recent literature also suggests that MGUS may contribute to cardiovascular disease (CVD) risk independent of malignant progression [28]. Studies have reported an increased incidence of both arterial and venous thrombosis, as well as elevated CVD risk in MGUS populations [29,30,31]. Considering the high median age (73 years) of MGUS patients in this cohort, many likely had pre-existing cardiovascular comorbidities such as hypertension, dyslipidemia, obesity, or type 2 diabetes. Further prospective studies are needed to determine whether MGUS itself is an independent risk factor for cardiovascular outcomes and whether NT-proBNP may play a role in early detection.

In MM, NT-proBNP levels increased with advancing disease stage across the ISS, R-ISS, and R2-ISS staging systems. However, after multivariate adjustment, NT-proBNP no longer showed an independent association with disease stage, while B2M, LDH, creatinine, and albumin remained significant predictors. These results support the interpretation that NT-proBNP is not an independent staging biomarker, but rather a surrogate for disease burden and systemic inflammation.

Serum B2M and albumin have long been established as key prognostic markers in MM and form the basis of the ISS staging system [15]. The inverse association between NT-proBNP and serum albumin, an acute-phase reactant, suggests that systemic inflammation may trigger cardiac stress. The associations between NT-proBNP and B2M, LDH, creatinine, and albumin imply a mechanistic link between inflammatory burden, renal dysfunction, and cardiac biomarker elevation. NT-proBNP is known to be upregulated in response to myocardial stress, and inflammation itself may stimulate NT-proBNP release from cardiomyocytes [32]. Markers such as creatinine, B2M, and albumin are also reflective of disease severity and tumor–host interactions [33]. These findings are consistent with those of Abe et al. [34], who reported that NT-proBNP was associated with both myeloma-related renal dysfunction and left ventricular diastolic dysfunction—highlighting NT-proBNP’s potential as a composite marker for both cardiac and renal impairment in MM.

Several limitations must be considered in interpreting this study. First, as a retrospective analysis based on existing clinical records, the findings may be subject to bias and confounding, and prospective validation is required. Second, the relatively small number of MGUS patients limited subgroup comparisons and statistical power. Third, incomplete cytogenetic data precluded the evaluation of relationships between NT-proBNP levels and specific genomic alterations. These limitations underscore the need for prospective, large-scale studies to further delineate the clinical utility of NT-proBNP in plasma cell dyscrasias.

## 5. Conclusions

In this retrospective analysis of patients with MGUS and MM, NT-proBNP was not associated with established MGUS risk factors, suggesting limited utility in risk stratification at the premalignant stage. However, in MM, NT-proBNP levels showed significant associations with surrogate markers of disease burden—including B2M, LDH, creatinine, and albumin—though not independently with disease stage. These findings suggest that NT-proBNP reflects systemic disease activity and organ involvement rather than serving as a direct marker of disease severity or progression. In MGUS, the elevation of NT-proBNP may be influenced more by renal function and subclinical cardiovascular comorbidities than by clonal plasma cell burden. While elevated NT-proBNP in MGUS could potentially indicate early cardiac involvement, such as occult amyloidosis, our data did not show a clear relationship with MGUS risk stratification. Given the known cardiovascular risks associated with both MGUS and MM, NT-proBNP may serve as a useful adjunct marker for assessing overall disease burden and organ function, particularly in older patients with comorbidities. However, its role as a biomarker for disease progression or risk stratification remains limited. Further prospective studies incorporating comprehensive cardiac, renal, and genomic assessments are needed to clarify the prognostic utility of NT-proBNP across the spectrum of plasma cell disorders and to explore its potential role in identifying patients at risk for subclinical organ involvement or adverse cardiovascular outcomes.

## Figures and Tables

**Table 1:** Characteristics of study population: 121 patients with monoclonal gammopathy of undetermined significance and 472 patients with multiple myeloma.

Characteristics	Overall (N = 593)		MGUS (N = 121)		MM (N = 472)		*p*-Value
Age (year)	71	[65, 77]	73	[69, 78]	70	[64, 76]	<0.001
≥70 years, N(%)	335	(56.5)	86	(71.1)	249	(52.8)	<0.001
Sex (Male, N(%))	316	(53.3)	86	(71.1)	230	(48.7)	<0.001
NT-proBNP (pg/mL)	335	[109, 1340]	421	[98, 2753]	320.5	[113.7, 1104.5]	0.168
≥300 pg/mL, N(%)	312	(52.6)	68	(56.2)	244	(51.7)	0.376
M-protein > 1.5 g/dL	264	(60.3)	22	(18.2)	242	(76.3)	<0.001
Non-IgG isotype	129	(32.2)	28	(23.1)	101	(36.1)	0.011
β2-microglobulin (mg/L)	4.4	[2.9, 8.1]	3.43	[2.18, 6.35]	4.72	[3.04, 8.69]	<0.001
Abnormal FLC ratio, N(%)	347	(79.2)	53	(43.8)	294	(92.7)	<0.001
Albumin (g/dL)	3.5	[3.1, 4]	3.6	[3.1, 4.2]	3.5	[3.1, 4]	0.331
Creatinine (mg/dL)	1.02	[0.78, 1.53]	1.09	[0.83, 1.62]	1	[0.77, 1.5]	0.128
≥2 mg/dL, N(%)	106	(17.9)	22	(18.2)	84	(17.8)	0.921
LDH (U/L)	387	[318, 481]	436	[377, 533]	375.5	[306.5, 468]	<0.001
Hgb (g/dL)	10	[8.7, 11.8]	11.4	[10.1, 13.3]	9.7	[8.6, 11.3]	<0.001
PLT (10^3^/μL)	199	[154.5, 252.5]	219	[177, 290]	193	[148, 248]	<0.001
Cytogenetics, N(%)							
del17p *	19	(3.2)	-		19	(4)	0.019
t(4; 14)	45	(7.6)	2	(1.7)	43	(9.1)	0.006
t(14; 16) *	5	(0.8)	-		5	(1.1)	0.589
1q	157	(26.5)	3	(2.5)	154	(32.6)	<0.001

* Fisher’s exact test. Data are n (%), or median [interquartile range] values. Abbreviations: NT-proBNP, N-terminal pro-B-type natriuretic peptide; M-protein, monoclonal protein; FLC ratio, free light chain ratio; LDH, lactate dehydrogenase; Hgb, hemoglobin; PLT, platelet.

**Table 2:** Characteristics of monoclonal gammopathy of undetermined significance (MGUS) according to Mayo MGUS risk stratification.

Characteristics	Low Risk (N = 47)		Low-Intermediate Risk (N = 47)		High-Intermediate Risk (N = 25)		High Risk (N = 2)		*p*-Value
Age (year)	74	[70, 79]	73	[69, 77]	73	[66, 80]	80.5	[72, 89]	0.472
Sex (Male, N(%))	35	(74.5)	36	(76.6)	13	(52)	2	(100)	0.126
NT-proBNP (pg/mL)	650.4	[128, 3128]	346	[84, 2493]	384	[70, 2903]	1411.5	[70, 2753]	0.476
M-protein > 1.5 g/dL	0	(0)	10	(21.3)	10	(40)	2	(100)	<0.001
Non-igG isotype, N(%)	0	(0)	10	(21.3)	16	(64)	2	(100)	<0.001
β2-microglobulin (mg/L)	3.43	[2.35, 7.04]	3.68	[1.89, 5.02]	3.36	[2.29, 7.35]	2.91	[2.91, 2.91]	0.777
Abnormal FLC ratio, N(%)	0	(0)	27	(57.5)	24	(96)	2	(100)	<0.001
Albumin (g/dL)	3.3	[2.8, 4.1]	3.9	[3.3, 4.3]	3.7	[3.5, 4.1]	3.2	[3.1, 3.3]	0.14
Creatinine (mg/dL)	1.1	[0.87, 1.7]	1.09	[0.88, 1.66]	1	[0.73, 1.56]	1.11	[0.82, 1.4]	0.778
≥2 mg/dL, N(%)	8	(17)	9	(19.2)	5	(20)	0	(0)	0.97
LDH (U/L)	436	[381, 539]	441	[345, 540]	428	[380, 529]	510	[225, 795]	0.982
Hgb (g/dL)	11.3	[9.9, 13.4]	11.5	[10.1, 13.2]	11.8	[10.5, 13.6]	11.6	[11.2, 12]	0.739
PLT (10^3^/μL)	208	[169, 285]	215	[170, 295]	242	[195, 290]	284.5	[198, 371]	0.446
Cytogenetics, N(%)									
del17p	0	(0)	0	(0)	0	(0)	0	(0)	-
t(4; 14) *	0	(0)	0	(0)	1	(4)	1	(50)	0.007
t(14; 16)	0	(0)	0	(0)	0	(0)	0	(0)	-
1q *	0	(0)	1	(2.1)	1	(4)	1	(50)	0.042

* Fisher’s exact test. Data are n (%), or median [interquartile range] values. Abbreviations: NT-proBNP, N-terminal pro-B-type natriuretic peptide; M-protein, monoclonal protein; FLC ratio, free light chain ratio; LDH, lactate dehydrogenase; Hgb, hemoglobin; PLT, platelet.

**Table 3:** Factors associated with NT-proBNP levels in patients with MGUS based on regression analysis.

Variables	Univariate				Multivariate			
	Standard Coeff.	Coeff.	SE	*p*-Value	Standard Coeff.	Coeff.	SE	*p*-Value
Age (year)	0.05	36.17	72.18	0.617	0.07	59.55	53.36	0.267
Sex (ref: female)	0.01	132.47	1354.96	0.922	−0.1	−1545.57	998.84	0.125
Abnormal FLC ratio (ref: normal)	−0.05	−697.42	1236.68	0.574	−0.01	−153.04	861.86	0.859
M-protein (ref: ≤1.5 g/dL)	0.01	202.71	1592.83	0.899	−0.0006	−10.47	1147.05	0.993
Non-IgG (ref: IgG type)	−0.05	−776.08	1455.09	0.595	−0.06	−984.05	1008.77	0.332
β2-microglobulin (mg/L)	0.73	738.07	66.29	<0.001	0.25	253.67	105.44	0.018
Albumin (g/dL)	−0.3	−2716.92	792.16	0.001	−0.14	−1354.73	716.45	0.062
LDH (U/L)	0.09	4	4.27	0.351	−0.08	−3.69	3.38	0.2781
Creatinine (mg/dL)	0.71	2502.5	229.71	<0.001	0.56	2113.92	362.36	<0.001
Hgb (g/dL)	−0.35	−1061.67	264.7	<0.001	0.005	14.46	238.88	0.952
PLT (10^3^/μL)	−0.06	−4.55	6.83	0.507	−0.08	−6.44	5.2	0.219

Adjusted R^2^ in multiple model is 0.621. Abbreviations: NT-proBNP, N-terminal pro-B-type natriuretic peptide; FLC ratio, free light chain ratio; M-protein, monoclonal protein; LDH, lactate dehydrogenase; Hgb, hemoglobin; PLT, platelet; coeff, coefficient.

**Table 4:** Characteristics of multiple myeloma (MM) according to MM risk stratifications.

Characteristics	ISS							R-ISS						
	I		II		III		*p*-Value	I		II		III		*p*-Value
	(N = 113)		(N = 152)		(N = 207)			(N = 76)		(N = 272)		(N = 124)		
Age (year)	66	[61, 72]	70	[65, 78]	72	[66, 77]	<0.001	66.5	[62, 71]	70	[64, 76]	73	[66.5, 77]	<0.001
Sex (Male, N(%))	66	(58.4)	66	(43.4)	98	(47.3)	0.047	48	(63.2)	124	(45.6)	58	(46.8)	0.022
NT-proBNP (pg/mL)	87.8	[70, 200]	258.2	[106.9, 680]	734	[285, 2228]	<0.001	81.9	[70, 174.5]	292.7	[120.3, 916.7]	882	[326.4, 2470]	<0.001
M-protein > 1.5 g/dL	41	(69.5)	89	(84.8)	112	(73.2)	0.039	26	(74.3)	152	(80)	64	(69.6)	0.147
Non-igG, N(%)	15	(29.4)	24	(25.5)	62	(45.9)	0.004	6	(20)	57	(33.9)	38	(46.3)	0.024
β2-microglobulin (mg/L)	2.34	[1.88, 2.97]	3.89	[3.21, 4.54]	9.28	[7.02, 14.66]	<0.001	2.32	[1.86, 2.89]	4.26	[3.2, 6.34]	9.55	[7.21, 18.13]	<0.001
Abnormal FLC ratio, N(%)	56	(94.9)	98	(93.3)	140	(91.5)	0.665	34	(97.1)	175	(92.1)	85	(92.4)	0.566
Albumin (g/dL)	4	[3.7, 4.3]	3.3	[3, 3.6]	3.4	[3, 3.8]	<0.001	4	[3.7, 4.3]	3.4	[3, 3.85]	3.4	[3, 3.8]	<0.001
Creatinine (mg/dL)	0.79	[0.63, 0.98]	0.85	[0.71, 1.10]	1.58	[1.04, 3.24]	<0.001	0.8	[0.65, 0.98]	0.96	[0.75, 1.3]	1.8	[1.065, 3.53]	<0.001
≥2 mg/dL, N(%)	0	(0)	3	(2)	81	(39.1)	<0.001	0	(0)	28	(10.3)	56	(45.2)	<0.001
LDH (U/L)	368	[309, 429]	352.5	[288.5, 456]	396	[318, 512]	0.008	343.5	[291.5, 387]	357	[292, 441]	482	[366.5, 592]	<0.001
Hgb (g/dL)	11.9	[10.2, 13.2]	9.6	[8.6, 10.9]	9	[8, 10.1]	<0.001	11.9	[10.2, 13]	9.5	[8.55, 10.8]	9.15	[7.9, 10.3]	<0.001
PLT (10^3^/μL)	216.5	[175.5, 265]	208.5	[158.5, 257]	168	[128, 223]	<0.001	219	[183, 264]	204	[153, 251.5]	160	[123.5, 205.5]	<0.001
Cytogenetics, N(%)														
del17p	1	(0.9)	9	(5.9)	9	(4.4)	0.113	0	(0)	10	(3.7)	9	(7.3)	0.025 *
t(4; 14)	9	(8)	17	(11.2)	17	(8.2)	0.557	0	(0)	26	(9.6)	17	(13.7)	0.004
t(14; 16)	0	(0)	0	(0)	5	(2.4)	0.059 *	0	(0)	0	(0)	5	(4)	0.002 *
1q	23	(20.4)	56	(36.8)	75	(36.2)	0.006	0	(0)	79	(29)	75	(60.5)	<0.001
**Characteristics**	**R2-ISS**													
	**I**			**II**			**III**			**IV**			***p*-Value**	
	**(N = 76)**			**(N = 94)**			**(N = 259)**			**(N = 43)**				
Age (year)	66.5	[62, 71]		69	[60, 75]		72	[66, 78]		70	[61, 77]		<0.001	
Sex (Male, N(%))	48	(63.2)		39	(41.5)		123	(47.5)		20	(46.5)		0.036	
NT-proBNP (pg/mL)	81.9	[70, 174.5]		140.3	[72, 353]		548	[223, 1685]		581	[227, 1800]		<0.001	
M-protein > 1.5 g/dL	26	(74.3)		48	(80)		146	(76.4)		22	(71)		0.796	
Non-IgG, N(%)	6	(20)		16	(31.4)		65	(37.4)		14	(56)		0.041	
β2-microglobulin (mg/L)	2.32	[1.86, 2.89]		3.32	[2.47, 4.14]		6.85	[4.29, 11.02]		8.78	[6.49, 17.86]		<0.001	
Abnormal FLC ratio, N(%)	34	(97.1)		57	(95)		173	(90.6)		30	(96.8)		0.465	
Albumin (g/dL)	4	[3.7, 4.3]		3.45	[3.1, 3.9]		3.4	[3, 3.8]		3.3	[3, 3.6]		<0.001	
Creatinine (mg/dL)	0.8	[0.645, 0.98]		0.81	[0.63, 1.01]		1.15	[0.85, 1.99]		1.26	[0.9, 3.51]		<0.001	
≥2 mg/dL, N(%)	0	(0)		2	(2.1)		64	(24.7)		18	(41.9)		<0.001	
LDH (U/L)	343.5	[291.5, 387]		360.5	[299, 418]		380	[306, 491]		527	[393, 600]		<0.001	
Hgb (g/dL)	11.9	[10.2, 13]		10.1	[9.1, 11.6]		9.2	[8.2, 10.5]		8.8	[8.3, 10.2]		<0.001	
PLT (10^3^/μL)	219	[183, 264]		224	[175, 285]		176	[136, 235]		164	[126, 202]		<0.001	
Cytogenetics, N(%)														
del17p	0	(0)		0	(0)		10	(3.9)		9	(20.9)		<0.001	
t(4; 14)	0	(0)		5	(5.3)		20	(7.7)		18	(41.9)		<0.001	
t(14; 16)	0	(0)		0	(0)		5	(1.9)		0	(0)		0.443 *	
1q	0	(0)		13	(13.8)		101	(39)		40	(93)		<0.001	

* Fisher’s exact test. Data are n (%), or median [interquartile range] values. Abbreviations: NT-proBNP, N-terminal pro-B-type natriuretic peptide; M-protein, monoclonal protein; FLC ratio, free light chain ratio; LDH, lactate dehydrogenase; Hgb, hemoglobin; PLT, platelet.

**Table 5:** Factors associated with NT-proBNP levels in patients with MM based on regression analysis.

Variables	Univariate			Multivariate								
				Model 1			Model 2			Model 3		
	Coeff.	SE	*p*-Value	Coeff.	SE	*p*-Value	Coeff.	SE	*p*-Value	Coeff.	SE	*p*-Value
Age (year)	40.07	20.56	0.052	13.02	19.66	0.508	11.62	19.62	0.554	5.98	19.66	0.761
Sex (ref: female)	−720	395.93	0.07	−712.88	389.69	0.068	−709.84	389.33	0.069	−725.94	389.31	0.063
ISS (ref = I)												
II	198.07	523.3	0.705	−776.54	550.43	0.159						
III	2020.32	492.77	<0.001	−253.34	630.08	0.688						
R-ISS (ref = I)												
II	599	545.8	0.273				−749.27	563.78	0.185			
III	2633.67	612.82	<0.001				−497.62	716.42	0.488			
R2-ISS (ref = I)												
II	−35.07	655.53	0.957							−1044.34	628.14	0.097
III	1603.36	554.38	0.004							−440	600.27	0.464
IV	1803.01	810.91	0.027							−1998.43	880.14	0.024
β2-microglobulin (mg/L)	172.8	24.14	<0.001	60.87	36.73	0.098	69.08	35.32	0.051	80.39	34.83	0.021
Albumin (g/dL)	−1116.12	305.46	<0.001	−1326.31	326.04	<0.001	−1301.38	319.09	<0.001	−1339.05	317.75	<0.001
LDH (U/L)	5.14	0.77	<0.001	3.91	0.75	<0.001	3.96	0.77	<0.001	4.42	0.78	<0.001
Creatinine (mg/dL)	748	100.78	<0.001	511.32	138.5	<0.001	505.63	138.23	<0.001	491.32	137.61	<0.001
Hgb (g/dL)	−154.47	94.63	0.103	114.39	110.21	0.300	110.47	106.84	0.302	119.54	107.33	0.266
PLT (10^3^/μL)	−0.7	2.22	0.752	3.38	2.13	0.113	3.18	2.13	0.136	3.04	2.13	0.153

Adjusted R^2^s are 0.222 in multivariate model 1, 0.196 in multivariate model 2 and 0.204 in multivariate model 3. Abbreviations: NT-proBNP, N-terminal pro-B-type natriuretic peptide; M-protein, monoclonal protein; LDH, lactate dehydrogenase; Hgb, hemoglobin; PLT, platelet; coeff, coefficient.

## Data Availability

The data presented in this study are available on request from the corresponding author.

## References

[1] Wiese S., Breyer T., Dragu A., Wakili R., Burkard T., Schmidt-Schweda S., Füchtbauer E.-M., Dohrmann U., Beyersdorf F., Radicke D. (2000). Gene Expression of Brain Natriuretic Peptide in Isolated Atrial and Ventricular Human Myocardium: Influence of Angiotensin II and Diastolic Fiber Length. Circulation.

[2] Volpe M., Rubattu S., Burnett J. (2014). Natriuretic Peptides in Cardiovascular Diseases: Current Use and Perspectives. Eur. Heart J..

[3] Pavo N., Raderer M., Hülsmann M., Neuhold S., Adlbrecht C., Strunk G., Goliasch G., Gisslinger H., Steger G.G. (2015). Cardiovascular Biomarkers in Patients with Cancer and Their Association with All-Cause Mortality. Heart.

[4] Andreu A., Guglin M. (2012). Exaggerated NT-proBNP Production in Patients with Hematologic Malignancies: A Case Series. Congest. Heart Fail..

[5] Burjonroppa S.C., Tong A.T., Xiao L.-C., Johnson M.M., Yusuf S.W., Lenihan D.J. (2007). Cancer Patients with Markedly Elevated B-Type Natriuretic Peptide May Not Have Volume Overload. Am. J. Clin. Oncol..

[6] Chovanec J., Chovanec J., Chovanec M., Mego M. (2023). Levels of NT-proBNP in Patients with Cancer. Oncol. Lett..

[7] Milani P., Vincent Rajkumar S., Merlini G., Kumar S., Gertz M.A., Palladini G., Lacy M.Q., Buadi F.K., Hayman S.R., Leung N. (2016). N-terminal Fragment of the type-B Natriuretic Peptide (NT-proBNP) Contributes to a Simple New Frailty Score in Patients with Newly Diagnosed Multiple Myeloma. Am. J. Hematol..

[8] Pavo N., Cho A., Wurm R., Strunk G., Krauth M., Agis H., Hülsmann M. (2018). NT-proBNP Is Associated with Disease Severity in Multiple Myeloma. Eur. J. Clin. Investig..

[9] Go R.S., Rajkumar S.V. (2018). How I Manage Monoclonal Gammopathy of Undetermined Significance. Blood.

[10] Rajkumar S.V., Dimopoulos M.A., Palumbo A., Blade J., Merlini G., Mateos M.-V., Kumar S., Hillengass J. (2014). International Myeloma Working Group Updated Criteria for the Diagnosis of Multiple Myeloma. Lancet Oncol..

[11] Mangiacavalli S., Milani P., Cartia C.S., Varettoni M., Palumbo M., Basset M., Masoni V., Nuvolone M., Foli A., Ferretti V.V. (2025). Feasibility of a Biomarker-Based Screening for Pre-Symptomatic AL Amyloidosis in Patients with Intermediate/High-Risk MGUS. Am. J. Hematol..

[12] Bellavia D., Pellikka P.A., Al-Zahrani G.B., Abraham T.P., Dispenzieri A., Miyazaki C., Lacy M., Scott C.G., Oh J.K., Miller F.A. (2010). Independent Predictors of Survival in Primary Systemic (Al) Amyloidosis, including cardiac biomarkers and left ventricular strain imaging: An observational cohort study. J. Am. Soc. Echocardiogr..

[13] Kyle R.A., Larson D.R., Therneau T.M., Dispenzieri A., Kumar S., Cerhan J.R., Rajkumar S.V. (2018). Long-Term Follow-up of Monoclonal Gammopathy of Undetermined Significance. N. Engl. J. Med..

[14] Rajkumar S.V., Kyle R.A., Buadi F.K. (2010). Advances in the Diagnosis, Classification, Risk Stratification, and Management of Monoclonal Gammopathy of Undetermined Significance. Mayo Clin. Proc..

[15] Greipp P.R., Miguel J.S., Durie B.G.M., Crowley J.J., Barlogie B., Bladé J., Boccadoro M., Child J.A., Avet-Loiseau H., Kyle R.A. (2005). International Staging System for Multiple Myeloma. J. Clin. Oncol..

[16] Palumbo A., Avet-Loiseau H., Oliva S., Lokhorst H.M., Goldschmidt H., Rosinol L., Richardson P., Caltagirone S. (2015). Revised International Staging System for Multiple Myeloma. J. Clin. Oncol..

[17] D’Agostino M., Cairns D.A., Lahuerta J.J., Wester R., Bertsch U., Waage A., Zamagni E., Mateos M.-V., Dall’Olio D. (2022). Second Revision of the International Staging System (R2-ISS) for Overall Survival in Multiple Myeloma. J. Clin. Oncol..

[18] Awada H., Thapa B., Awada H., Dong J., Gurnari C., Hari P., Dhakal B. (2021). A Comprehensive Review of the Genomics of Multiple Myeloma. Cells.

[19] Kaur G., Jena L., Gupta R., Farswan A., Gupta A., Sriram K. (2022). Correlation of Changes in Subclonal Architecture with Progression in the MMRF CoMMpass Study. Transl. Oncol..

[20] Walker B.A., Wardell C.P., Melchor L., Brioli A., Johnson D.C., Kaiser M.F., Mirabella F., Lopez-Corral L., Humphray S., Murray L. (2013). Intraclonal Heterogeneity Is a Critical Early Event in the Development of Myeloma. Leukemia.

[21] Rajkumar S.V. (2022). Multiple Myeloma: 2022 Update on Diagnosis, Risk Stratification, and Management. Am. J. Hematol..

[22] Jang M.-A. (2024). Genomic Technologies for Detecting Structural Variations in Hematologic Malignancies. Blood Res..

[23] Bataille R., Durie B.G., Grenier J., Sany J. (1986). Prognostic Factors and Staging in Multiple Myeloma: A Reappraisal. J. Clin. Oncol..

[24] Perosa F., Luccarelli G., Prete M., Ferrone S., Dammacco F. (1999). Increased Serum Levels of Beta2m-Free HLA Class I Heavy Chain in Multiple Myeloma. Br. J. Haematol..

[25] Christianson G.J., Brooks W., Vekasi S., Manolfi E.A., Niles J., Roopenian S.L., Roths J.B., Rothlein R., Roopenian D.C. (1997). Beta 2-Microglobulin-Deficient Mice Are Protected from Hypergammaglobulinemia. J. Immunol..

[26] Hamada R., Kikunaga K., Kaneko T., Okamoto S., Tomotsune M., Uemura O., Kamei K., Wada N., Matsuyama T., Ishikura K. (2023). Urine Alpha 1-Microglobulin-to-Creatinine Ratio and Beta 2-Microglobulin-to-Creatinine Ratio for Detecting CAKUT in Children. Pediatr. Nephrol..

[27] Hansson E., Wegman D.H., Wesseling C., Glaser J., Schlader Z.J., Wijkström J., Jakobsson K. (2022). Markers of Kidney Tubular and Interstitial Injury and Function among Sugarcane Workers with Cross-Harvest Serum Creatinine Elevation. Occup. Environ. Med..

[28] Tentolouris A., Ntanasis-Stathopoulos I., Gavriatopoulou M., Andreadou I., Terpos E. (2023). Monoclonal Gammopathy of Undetermined Cardiovascular Significance; Current Evidence and Novel Insights. J. Cardiovasc. Dev. Dis..

[29] Gkalea V., Fotiou D., Dimopoulos M.A., Kastritis E. (2023). Monoclonal Gammopathy of Thrombotic Significance. Cancers.

[30] Schwartz B., Schou M., Ruberg F.L., Rucker D., Choi J., Siddiqi O., Monahan K., Køber L., Gislason G., Torp-Pedersen C. (2022). Cardiovascular Morbidity in Monoclonal Gammopathy of Undetermined Significance: A Danish Nationwide Study. JACC Cardio Oncol..

[31] Kapoor P., Rajkumar S.V. (2022). Cardiovascular Associations with Monoclonal Gammopathy of Undetermined Significance: Real or Coincidental?. JACC Cardio Oncol..

[32] Vila G., Resl M., Stelzeneder D., Struck J., Maier C., Riedl M., Hülsmann M., Pacher R., Luger A., Clodi M. (2008). Plasma NT-proBNP Increases in Response to LPS Administration in Healthy Men. J. Appl. Physiol..

[33] Kim J.E., Yoo C., Lee D.H., Kim S.-W., Lee J.-S., Suh C. (2010). Serum Albumin Level Is a Significant Prognostic Factor Reflecting Disease Severity in Symptomatic Multiple Myeloma. Ann. Hematol..

[34] Abe Y., Kobayashi T., Usui Y., Narita K., Kobayashi H., Kitadate A., Miura D., Takeuchi M., Matsue K. (2019). N-Terminal pro-Brain Natriuretic Peptide Reflects Both Left Ventricular Diastolic Dysfunction and Myeloma-Related Renal Insufficiency and Robustly Predicts Mortality in Patients with Symptomatic Multiple Myeloma. Oncotarget.

